# (*E*)-1-(Naphthalen-1-yl)-3-(1-phenyl-1*H*-pyrazol-4-yl)prop-2-en-1-one

**DOI:** 10.1107/S1600536811035124

**Published:** 2011-09-03

**Authors:** Abdullah M. Asiri, Abdulrahman O. Al-Youbi, Hassan M. Faidallah, Khalid A. Alamry, Seik Weng Ng

**Affiliations:** a Chemistry Department, Faculty of Science, King Abdulaziz University, PO Box 80203 Jeddah, Saudi Arabia; b Center of Excellence for Advanced Materials Research, King Abdulaziz University, PO Box 80203 Jeddah, Saudi Arabia; c Department of Chemistry, University of Malaya, 50603 Kuala Lumpur, Malaysia

## Abstract

In the title mol­ecule, C_22_H_16_N_2_O, the phenyl ring is twisted slightly with respect to the plane of the central pyrazole ring [dihedral angle = 14.8 (2)°]; the central ring is connected to the naphthyl ring through a —CH=CH—C(=O)— fragment, whose C=C double bond has an *E* configuration. The pyrazole ring and naphthalene ring system are twisted by 46.3 (1)°. Weak inter­molecular C—H⋯O hydrogen bonds link the mol­ecules, forming supra­molecular chains running along the *a* axis. The crystal studied was a non-merohedral twin with a component ratio of 0.544 (2):0.456 (2).

## Related literature

For related structures; see: Diánez & López-Castro (1990 ▶); Jones *et al.* (1984 ▶). For the synthesis, see: Finar (1961 ▶); Finar & Lord (1959 ▶); Jones *et al.* (1984 ▶).
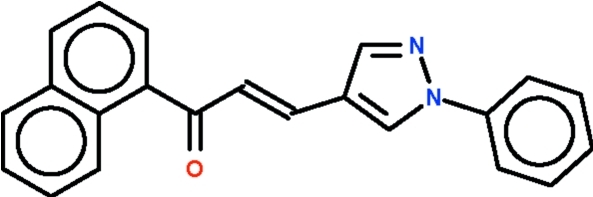


## Experimental

### 

#### Crystal data

C_22_H_16_N_2_O
*M* *_r_* = 324.37
Monoclinic, 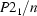

*a* = 5.8457 (6) Å
*b* = 10.322 (2) Å
*c* = 26.626 (2) Å
β = 92.322 (9)°
*V* = 1605.3 (4) Å^3^
*Z* = 4
Mo *K*α radiation
μ = 0.08 mm^−1^
*T* = 100 K
0.25 × 0.10 × 0.10 mm

#### Data collection

Agilent SuperNova Dual diffractometer with an Atlas detector
Absorption correction: multi-scan (*CrysAlis PRO*; Agilent, 2010 ▶) *T*_min_ = 0.980, *T*_max_ = 0.992
3824 measured reflections
3825 independent reflections
2494 reflections with *I* > 2σ(*I*)
*R*_int_ = 0.105

#### Refinement

*R*[*F* ^2^ > 2σ(*F* ^2^)] = 0.060
*wR*(*F* ^2^) = 0.140
*S* = 0.96
3825 reflections
227 parameters
H-atom parameters constrained
Δρ_max_ = 0.31 e Å^−3^
Δρ_min_ = −0.34 e Å^−3^

### 

Data collection: *CrysAlis PRO* (Agilent, 2010 ▶); cell refinement: *CrysAlis PRO*; data reduction: *CrysAlis PRO*; program(s) used to solve structure: *SHELXS97* (Sheldrick, 2008 ▶); program(s) used to refine structure: *SHELXL97* (Sheldrick, 2008 ▶); molecular graphics: *X-SEED* (Barbour, 2001 ▶); software used to prepare material for publication: *publCIF* (Westrip, 2010 ▶).

## Supplementary Material

Crystal structure: contains datablock(s) global, I. DOI: 10.1107/S1600536811035124/xu5313sup1.cif

Structure factors: contains datablock(s) I. DOI: 10.1107/S1600536811035124/xu5313Isup2.hkl

Supplementary material file. DOI: 10.1107/S1600536811035124/xu5313Isup3.cml

Additional supplementary materials:  crystallographic information; 3D view; checkCIF report

## Figures and Tables

**Table 1:** Hydrogen-bond geometry (Å, °)

*D*—H⋯*A*	*D*—H	H⋯*A*	*D*⋯*A*	*D*—H⋯*A*
C9—H9⋯O1^i^	0.95	2.46	3.397 (4)	167

Symmetry code: (i) 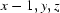.

**Fig. 1.**
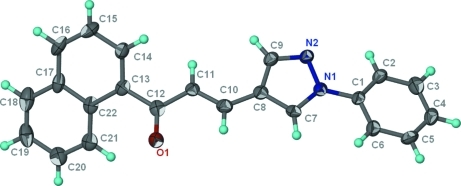
Thermal ellipsoid plot (Barbour, 2001) of C22H16N2O at the 70% probability level; hydrogen atoms are drawn as spheres of arbitrary radius.

## supplementary crystallographic information

### Comment

The hydrogen of the acetyl group of 1-acetylnaphthalene (as well as that of similar ketones) is relatively acidic, and can be abstracted by a strong base. In the present study, the resulting carbanion is used for carbon-carbon double-bond synthesis to extend the nature of the substitutent at the 4-position of 1-phenylpyrazole-4-carboxaldehyde by using a similar procedure for synthesizing the 1-phenyl-3-(1-phenyl-1*H*-pyrazol-4-yl)prop-2-en-1-one (Finar, 1961; Finar & Lord, 1959). In the C_22_H_16_N_2_O molecule (Scheme I), the phenyl ring is slightly twisted with respect to the central pyrazole; the central ring is connected to the naphthyl ring through the –CH–CH–C(═O)– fragment, whose C–C double-bond is of an *E*-configuration (Fig. 1). The pyrazole and naphthalene rings are twisted by 46.3 (1) °. There are only few crystal structure reports of 4-substituted 1-phenylpyrazoles (Diánez López-Castro, 1990; Jones *et al.*, 1984).

### Experimental

1-Phenylpyrazole-4-carboxaldehyde (0.01 mol) in ethanol (20 ml) was added to a 1-acetylnaphthalene (0.01 mol) in dissolved in 20% ethanolic potassium hydroxide (20 ml). The mixture was stirred for 6 h. The mixture was then poured into water (200 ml). The precipitated product was collected by filtration, washed with water, dried and recrystallized from ethanol; m.p. 389–391 K.

### Refinement

Carbon- and nitrogen-bound H-atoms were placed in calculated positions [C–H 0.95, *U*_iso_(H) 1.2*U*_eq_(C)] and were included in the refinement in the riding model approximation.

The crystal is a non-merohedral twin; integration of the diffraction spots gave a ratio of 0.539: 0.461 for the 8472 reflections, most of which were overlapped. Of the isolated spots, the *R*_int_ of the major component was 0.017 and that of the minor component was 0.021. The ratio refined to 0.544 (2): 0.456.

Omitted were (1 - 5 5), (-4 - 5 -8) and (-4 - 6 -8).

### Crystal data

**Table:**

C_22_H_16_N_2_O	*F*(000) = 680
*M**_r_* = 324.37	*D*_x_ = 1.342 Mg m^−^^3^
Monoclinic, *P*2_1_/*n*	Mo *K*α radiation, λ = 0.71073 Å
Hall symbol: -P 2yn	Cell parameters from 1183 reflections
*a* = 5.8457 (6) Å	θ = 2.5–27.5°
*b* = 10.322 (2) Å	µ = 0.08 mm^−^^1^
*c* = 26.626 (2) Å	*T* = 100 K
β = 92.322 (9)°	Prism, colorless
*V* = 1605.3 (4) Å^3^	0.25 × 0.10 × 0.10 mm
*Z* = 4	

### Data collection

**Table:**

Agilent SuperNova Dual diffractometer with an Atlas detector	3825 independent reflections
Radiation source: SuperNova (Cu) X-ray Source	2494 reflections with *I* > 2σ(*I*)
Mirror	*R*_int_ = 0.105
Detector resolution: 10.4041 pixels mm^-1^	θ_max_ = 27.5°, θ_min_ = 2.5°
ω scans	*h* = −5→7
Absorption correction: multi-scan (*CrysAlis PRO*; Agilent, 2010)	*k* = −13→13
*T*_min_ = 0.980, *T*_max_ = 0.992	*l* = −34→33
3824 measured reflections	

### Refinement

**Table:**

Refinement on *F*^2^	Primary atom site location: structure-invariant direct methods
Least-squares matrix: full	Secondary atom site location: difference Fourier map
*R*[*F*^2^ > 2σ(*F*^2^)] = 0.060	Hydrogen site location: inferred from neighbouring sites
*wR*(*F*^2^) = 0.140	H-atom parameters constrained
*S* = 0.96	*w* = 1/[σ^2^(*F*_o_^2^) + (0.0432*P*)^2^] where *P* = (*F*_o_^2^ + 2*F*_c_^2^)/3
3825 reflections	(Δ/σ)_max_ = 0.001
227 parameters	Δρ_max_ = 0.31 e Å^−^^3^
0 restraints	Δρ_min_ = −0.34 e Å^−^^3^

### Fractional atomic coordinates and isotropic or equivalent isotropic displacement parameters (Å^2^)

**Table:**

	*x*	*y*	*z*	*U*_iso_*/*U*_eq_	
O1	1.1745 (4)	0.6128 (2)	0.63013 (9)	0.0283 (5)	
N1	0.4987 (4)	0.2604 (2)	0.49661 (10)	0.0202 (6)	
N2	0.3221 (4)	0.3061 (2)	0.52392 (10)	0.0229 (6)	
C1	0.4623 (5)	0.1552 (3)	0.46241 (11)	0.0213 (7)	
C2	0.2589 (5)	0.0852 (3)	0.46313 (12)	0.0243 (7)	
H2	0.1425	0.1097	0.4851	0.029*	
C3	0.2277 (6)	−0.0202 (3)	0.43169 (13)	0.0305 (8)	
H3	0.0889	−0.0679	0.4320	0.037*	
C4	0.3976 (6)	−0.0571 (3)	0.39953 (13)	0.0293 (8)	
H4	0.3767	−0.1305	0.3783	0.035*	
C5	0.5974 (6)	0.0145 (3)	0.39896 (13)	0.0303 (8)	
H5	0.7139	−0.0095	0.3769	0.036*	
C6	0.6297 (5)	0.1211 (3)	0.43018 (12)	0.0271 (7)	
H6	0.7669	0.1702	0.4293	0.032*	
C7	0.7000 (5)	0.3184 (3)	0.51010 (12)	0.0225 (7)	
H7	0.8446	0.3016	0.4964	0.027*	
C8	0.6552 (5)	0.4065 (3)	0.54748 (12)	0.0220 (7)	
C9	0.4192 (5)	0.3936 (3)	0.55371 (12)	0.0247 (7)	
H9	0.3375	0.4432	0.5771	0.030*	
C10	0.8217 (5)	0.4858 (3)	0.57469 (12)	0.0257 (7)	
H10	0.9746	0.4815	0.5640	0.031*	
C11	0.7837 (5)	0.5642 (3)	0.61324 (11)	0.0200 (7)	
H11	0.6309	0.5792	0.6227	0.024*	
C12	0.9745 (5)	0.6283 (3)	0.64149 (12)	0.0226 (7)	
C13	0.9159 (5)	0.7116 (3)	0.68505 (12)	0.0220 (7)	
C14	0.7203 (5)	0.7865 (3)	0.67969 (13)	0.0265 (7)	
H14	0.6237	0.7780	0.6503	0.032*	
C15	0.6629 (6)	0.8752 (3)	0.71719 (14)	0.0336 (8)	
H15	0.5319	0.9290	0.7124	0.040*	
C16	0.7945 (6)	0.8842 (3)	0.76025 (14)	0.0325 (8)	
H16	0.7533	0.9443	0.7853	0.039*	
C17	0.9905 (6)	0.8066 (3)	0.76865 (12)	0.0260 (7)	
C18	1.1253 (7)	0.8148 (3)	0.81331 (14)	0.0367 (9)	
H18	1.0838	0.8748	0.8384	0.044*	
C19	1.3144 (6)	0.7390 (3)	0.82175 (13)	0.0358 (9)	
H19	1.4046	0.7474	0.8521	0.043*	
C20	1.3740 (6)	0.6488 (3)	0.78517 (13)	0.0321 (8)	
H20	1.5023	0.5938	0.7914	0.038*	
C21	1.2500 (5)	0.6390 (3)	0.74068 (13)	0.0269 (7)	
H21	1.2941	0.5774	0.7164	0.032*	
C22	1.0581 (5)	0.7185 (3)	0.73024 (12)	0.0232 (7)	

### Atomic displacement parameters (Å^2^)

**Table:**

	*U*^11^	*U*^22^	*U*^33^	*U*^12^	*U*^13^	*U*^23^
O1	0.0241 (13)	0.0340 (13)	0.0269 (13)	−0.0001 (10)	0.0011 (10)	−0.0068 (10)
N1	0.0224 (13)	0.0212 (13)	0.0169 (13)	0.0039 (11)	−0.0013 (11)	0.0008 (11)
N2	0.0248 (14)	0.0259 (14)	0.0180 (13)	0.0068 (11)	−0.0010 (12)	−0.0021 (11)
C1	0.0264 (17)	0.0190 (15)	0.0182 (15)	0.0081 (13)	−0.0043 (14)	−0.0012 (13)
C2	0.0281 (17)	0.0203 (15)	0.0243 (17)	0.0061 (13)	0.0003 (15)	−0.0022 (14)
C3	0.0350 (19)	0.0213 (17)	0.034 (2)	0.0003 (15)	−0.0087 (17)	0.0008 (15)
C4	0.040 (2)	0.0213 (16)	0.0260 (18)	0.0082 (15)	−0.0087 (17)	−0.0038 (14)
C5	0.0303 (19)	0.0343 (18)	0.0262 (18)	0.0151 (15)	0.0015 (16)	−0.0036 (15)
C6	0.0308 (18)	0.0257 (16)	0.0243 (17)	0.0051 (14)	−0.0051 (15)	−0.0047 (14)
C7	0.0186 (15)	0.0247 (17)	0.0240 (18)	0.0050 (13)	−0.0031 (14)	0.0012 (14)
C8	0.0243 (16)	0.0208 (15)	0.0209 (17)	0.0049 (13)	−0.0018 (14)	0.0026 (13)
C9	0.0295 (18)	0.0253 (17)	0.0190 (17)	0.0091 (14)	−0.0010 (15)	−0.0015 (14)
C10	0.0234 (17)	0.0271 (17)	0.0263 (17)	0.0053 (14)	−0.0013 (14)	0.0013 (15)
C11	0.0207 (17)	0.0202 (15)	0.0189 (16)	0.0027 (12)	−0.0004 (14)	0.0022 (13)
C12	0.0293 (18)	0.0156 (15)	0.0227 (17)	−0.0010 (13)	−0.0017 (15)	0.0066 (13)
C13	0.0279 (17)	0.0151 (14)	0.0231 (17)	−0.0014 (13)	0.0036 (15)	0.0019 (13)
C14	0.0313 (19)	0.0242 (16)	0.0240 (17)	0.0035 (14)	−0.0009 (15)	−0.0048 (14)
C15	0.038 (2)	0.0252 (17)	0.039 (2)	0.0107 (15)	0.0073 (18)	−0.0052 (15)
C16	0.045 (2)	0.0214 (17)	0.032 (2)	0.0010 (15)	0.0120 (18)	−0.0088 (15)
C17	0.0391 (19)	0.0186 (16)	0.0206 (17)	−0.0092 (14)	0.0054 (16)	−0.0031 (13)
C18	0.059 (2)	0.0261 (18)	0.0253 (18)	−0.0112 (17)	0.0028 (19)	−0.0022 (15)
C19	0.054 (2)	0.0307 (19)	0.0216 (18)	−0.0105 (17)	−0.0101 (18)	0.0035 (16)
C20	0.037 (2)	0.0273 (18)	0.0319 (19)	−0.0010 (15)	−0.0035 (17)	0.0036 (15)
C21	0.0312 (18)	0.0214 (16)	0.0281 (18)	−0.0047 (13)	0.0027 (16)	0.0014 (14)
C22	0.0295 (18)	0.0165 (15)	0.0238 (16)	−0.0066 (13)	0.0036 (15)	0.0034 (13)

### Geometric parameters (Å, °)

**Table:**

O1—C12	1.230 (4)	C10—H10	0.9500
N1—C7	1.355 (4)	C11—C12	1.477 (4)
N1—N2	1.370 (3)	C11—H11	0.9500
N1—C1	1.428 (4)	C12—C13	1.494 (4)
N2—C9	1.316 (4)	C13—C14	1.383 (4)
C1—C6	1.373 (4)	C13—C22	1.436 (4)
C1—C2	1.392 (4)	C14—C15	1.405 (4)
C2—C3	1.380 (4)	C14—H14	0.9500
C2—H2	0.9500	C15—C16	1.358 (5)
C3—C4	1.391 (5)	C15—H15	0.9500
C3—H3	0.9500	C16—C17	1.408 (5)
C4—C5	1.383 (5)	C16—H16	0.9500
C4—H4	0.9500	C17—C18	1.402 (5)
C5—C6	1.387 (4)	C17—C22	1.436 (4)
C5—H5	0.9500	C18—C19	1.366 (5)
C6—H6	0.9500	C18—H18	0.9500
C7—C8	1.381 (4)	C19—C20	1.402 (4)
C7—H7	0.9500	C19—H19	0.9500
C8—C9	1.402 (4)	C20—C21	1.367 (5)
C8—C10	1.444 (4)	C20—H20	0.9500
C9—H9	0.9500	C21—C22	1.409 (4)
C10—C11	1.334 (4)	C21—H21	0.9500
			
C7—N1—N2	111.8 (2)	C10—C11—H11	119.4
C7—N1—C1	127.6 (3)	C12—C11—H11	119.4
N2—N1—C1	120.3 (2)	O1—C12—C11	121.5 (3)
C9—N2—N1	103.9 (2)	O1—C12—C13	121.1 (3)
C6—C1—C2	120.4 (3)	C11—C12—C13	117.4 (3)
C6—C1—N1	120.1 (3)	C14—C13—C22	120.4 (3)
C2—C1—N1	119.4 (3)	C14—C13—C12	117.1 (3)
C3—C2—C1	119.5 (3)	C22—C13—C12	122.4 (3)
C3—C2—H2	120.2	C13—C14—C15	120.7 (3)
C1—C2—H2	120.2	C13—C14—H14	119.6
C2—C3—C4	120.6 (3)	C15—C14—H14	119.6
C2—C3—H3	119.7	C16—C15—C14	120.0 (3)
C4—C3—H3	119.7	C16—C15—H15	120.0
C5—C4—C3	119.0 (3)	C14—C15—H15	120.0
C5—C4—H4	120.5	C15—C16—C17	121.7 (3)
C3—C4—H4	120.5	C15—C16—H16	119.2
C4—C5—C6	120.8 (3)	C17—C16—H16	119.2
C4—C5—H5	119.6	C18—C17—C16	121.7 (3)
C6—C5—H5	119.6	C18—C17—C22	118.8 (3)
C1—C6—C5	119.6 (3)	C16—C17—C22	119.5 (3)
C1—C6—H6	120.2	C19—C18—C17	121.9 (3)
C5—C6—H6	120.2	C19—C18—H18	119.1
N1—C7—C8	107.1 (3)	C17—C18—H18	119.1
N1—C7—H7	126.5	C18—C19—C20	119.2 (3)
C8—C7—H7	126.5	C18—C19—H19	120.4
C7—C8—C9	103.8 (3)	C20—C19—H19	120.4
C7—C8—C10	126.2 (3)	C21—C20—C19	120.9 (3)
C9—C8—C10	129.9 (3)	C21—C20—H20	119.6
N2—C9—C8	113.3 (3)	C19—C20—H20	119.6
N2—C9—H9	123.3	C20—C21—C22	121.2 (3)
C8—C9—H9	123.3	C20—C21—H21	119.4
C11—C10—C8	126.9 (3)	C22—C21—H21	119.4
C11—C10—H10	116.6	C21—C22—C17	117.9 (3)
C8—C10—H10	116.6	C21—C22—C13	124.4 (3)
C10—C11—C12	121.2 (3)	C17—C22—C13	117.5 (3)
			
C7—N1—N2—C9	0.7 (3)	O1—C12—C13—C14	142.2 (3)
C1—N1—N2—C9	175.1 (2)	C11—C12—C13—C14	−38.7 (4)
C7—N1—C1—C6	−15.4 (4)	O1—C12—C13—C22	−35.7 (4)
N2—N1—C1—C6	171.1 (3)	C11—C12—C13—C22	143.4 (3)
C7—N1—C1—C2	162.6 (3)	C22—C13—C14—C15	3.1 (5)
N2—N1—C1—C2	−10.9 (4)	C12—C13—C14—C15	−174.8 (3)
C6—C1—C2—C3	0.9 (5)	C13—C14—C15—C16	−2.9 (5)
N1—C1—C2—C3	−177.1 (3)	C14—C15—C16—C17	0.3 (5)
C1—C2—C3—C4	0.2 (5)	C15—C16—C17—C18	−179.4 (3)
C2—C3—C4—C5	−1.0 (5)	C15—C16—C17—C22	2.0 (5)
C3—C4—C5—C6	0.5 (5)	C16—C17—C18—C19	179.5 (3)
C2—C1—C6—C5	−1.3 (4)	C22—C17—C18—C19	−1.9 (5)
N1—C1—C6—C5	176.6 (3)	C17—C18—C19—C20	−1.1 (5)
C4—C5—C6—C1	0.6 (5)	C18—C19—C20—C21	2.2 (5)
N2—N1—C7—C8	−0.4 (3)	C19—C20—C21—C22	−0.3 (5)
C1—N1—C7—C8	−174.4 (3)	C20—C21—C22—C17	−2.6 (5)
N1—C7—C8—C9	0.0 (3)	C20—C21—C22—C13	−178.3 (3)
N1—C7—C8—C10	176.8 (3)	C18—C17—C22—C21	3.7 (4)
N1—N2—C9—C8	−0.7 (3)	C16—C17—C22—C21	−177.7 (3)
C7—C8—C9—N2	0.4 (4)	C18—C17—C22—C13	179.6 (3)
C10—C8—C9—N2	−176.2 (3)	C16—C17—C22—C13	−1.7 (4)
C7—C8—C10—C11	−174.8 (3)	C14—C13—C22—C21	174.9 (3)
C9—C8—C10—C11	1.1 (6)	C12—C13—C22—C21	−7.3 (5)
C8—C10—C11—C12	172.8 (3)	C14—C13—C22—C17	−0.8 (4)
C10—C11—C12—O1	0.8 (5)	C12—C13—C22—C17	177.0 (3)
C10—C11—C12—C13	−178.3 (3)		

### Hydrogen-bond geometry (Å, °)

**Table:**

*D*—H···*A*	*D*—H	H···*A*	*D*···*A*	*D*—H···*A*
C9—H9···O1^i^	0.95	2.46	3.397 (4)	167

Symmetry codes: (i) *x*−1, *y*, *z*.
